# Development and clinical application of a stability-indicating chromatography technique for the quantification of diazoxide

**DOI:** 10.1016/j.heliyon.2023.e20101

**Published:** 2023-09-13

**Authors:** Trusha J. Purohit, Don Laing, Christopher JD. McKinlay, Jane M. Alsweiler, Sara M. Hanning

**Affiliations:** a School of Pharmacy, The University of Auckland, Auckland, New Zealand; b Kidz First Neonatal Care, Counties Manukau Health, Auckland, New Zealand; c Department of Paediatrics: Child and Youth Health, The University of Auckland, Auckland, New Zealand; d Neonatal Care, Starship Hospital, Auckland, New Zealand

**Keywords:** Diazoxide, High-performance liquid chromatography, Plasma, Extemporaneous compounding, Protein precipitation

## Abstract

Diazoxide is a potential candidate for the treatment of transitional hypoglycaemia in infants. A clinical trial is currently underway to investigate whether low-dose oral diazoxide is beneficial for severe or recurrent transitional neonatal hypoglycaemia (the NeoGluCO Study, registration ANZCTR12620000129987). The present study aimed to develop and validate the parameters for quantifying diazoxide from neonatal plasma samples, and to assess the stability of extemporaneously prepared diazoxide suspensions to support the NeoGluCO Study. To determine the plasma concentration of diazoxide, a protein precipitation mediated extraction protocol was developed, which demonstrated >94% diazoxide extraction recoveries from all samples. The method was linear over the range of 0.2–40 μg/mL (R^2^ > 0.9994) with a limit of quantification of 0.2 μg/mL. Accuracy of the method was within 97–106% with relative standard deviation < 6% for all samples. Diazoxide-plasma samples were stable for up to three months at −20 °C and up to 48 h when stored in the auto-sampler. Samples were stable for up to two freeze-thaw cycles, with further cycles compromising stability of diazoxide in plasma. The developed method was applied to determine chemical stability of the extemporaneously prepared diazoxide suspensions. These were stable at both 2–8 °C and 25 °C/60% RH, with 98% of diazoxide remaining after 35 days in both storage conditions. Diazoxide was successfully quantified from plasma collected from six neonates enrolled in the NeoGluCO Study, using the developed protocol. Overall, an efficient and reproducible extraction protocol was developed and validated for the estimation of diazoxide from human plasma.

## 1 Introduction

Transitional neonatal hypoglycaemia occurs in ∼15% of newborn infants and is of importance because of its association with neurocognitive impairment and reduced educational attainment [1]. It results from inadequate neonatal hepatic glucose output after birth, which is thought to be primarily due to dysregulation of the pancreatic β-cell, leading to inappropriately high insulin secretion [2,3]. Infants with severe or recurrent hypoglycaemia are at highest risk of long-term adverse outcomes and new treatment approaches are needed for this group that target the underlying pathophysiology [4]. Diazoxide is one such potential treatment that acts on ATP-sensitive potassium channel causing hyperpolarization of the pancreatic β-cell, which in turn limits insulin release [5,6]. Advantages of diazoxide include oral formulation, good absorption, rapid onset of action and low cost [7]. In a recent randomised trial in small-for-gestational age infants, early use of diazoxide for transitional hypoglycaemia reduced time to euglycemia and establishment of full enteral bolus feeds, and duration of intravenous fluids [8]. However, further clinical evidence is needed to confirm these results and to assess the role of diazoxide in other infants with transitional hypoglycaemia [9]. In this study, extemporaneously compounded 10 mg/mL diazoxide oral liquid was evaluated for its stability and efficacy in the Neonatal Glucose Care Optimisation (NeoGluCO) Study (registration ANZCTR12620000129987).

The NeoGluCO Study is a double-blind, randomised-controlled trial of diazoxide versus placebo, to investigate whether use of diazoxide in neonates born at ≥35 weeks’ gestation with severe or recurrent transitional hypoglycaemia in the first week after birth reduces the time to resolve neonatal hypoglycaemia. The infants receive a loading dose of diazoxide 5 mg/kg followed by 1.5 mg/kg maintenance dose every 12 h, or an equal volume placebo. Diazoxide oral liquid 10 mg/mL was extemporaneously compounded from capsules using Ora Blend® SF (OB SF) as the suspending vehicle. The placebo consisted of OB SF, combined with a small amount of corn starch (4 g per 50 ml) to ensure similar appearance to the diazoxide suspension.

To our knowledge, only one study has investigated the chemical stability of diazoxide capsules suspended in an extemporaneously compounded oral suspension [10]. This study found that diazoxide capsules were stable in Oral Mix and Oral Mix SF vehicles for up to 90 days. However, in New Zealand, the commercial suspending vehicles Ora Blend® and Ora Blend® SF (OB SF) are subsidised and, therefore, are preferred for extemporaneous formulations. Oral Mix and Ora Mix SF are similar in composition to Ora Blend® and OB SF, but there are some differences that may influence suspension stability [11].

An important secondary outcome of the NeoGluCO Study is to measure plasma diazoxide concentration at 36 h after commencing the intervention (before the third maintenance dose). Kizu et al. proposed a population pharmacokinetic model for diazoxide in children with hyperinsulinaemic hypoglycaemia and found that diazoxide serum concentrations greater than 100 μg/mL were associated with a higher risk of hyperglycaemia [7]. It is unknown whether a similar threshold exists in neonates with transitional hypoglycaemia. Although a method for extracting diazoxide from plasma has been developed previously, extraction was found to be less than 85% [12].

The aim of this study was to 1) develop and validate a stability-indicating chromatography method for *in vitro* and *in vivo* quantification of diazoxide in human plasma; and 2) to evaluate the physicochemical stability of extemporaneously compounded 10 mg/mL diazoxide suspension. Data generated from this work is necessary to support the diazoxide formulation to be used in the NeoGluCO clinical trial, and to allow plasma diazoxide concentration to be quantified. It also has future application for enabling evidence-based and standardised extemporaneous compounding of diazoxide suspensions, which aligns with the mission and goals of the International Pharmaceutical Federation Paediatric Formulations focus group [13].

## 2 Material and methods

### 2.1 Materials

Diazoxide pharmacopoeial reference standard (purity >99%) was purchased from Sigma Aldrich (St Louis, MO, USA). Diazoxide suspensions were extemporaneously compounded using diazoxide capsules (Proglicem 100 mg, Merck Sharp and Dohme, Kenilworth, New Jersey, USA) and OB SF (Perrigo, Dublin, Ireland). Acetonitrile, methanol and potassium hydroxide pellets were purchased from Sigma Aldrich (St Louis, MO, USA). The solvents used for chromatographic analyses were high performance liquid chromatography (HPLC) grade and the Milli-Q water was obtained from Millipak (Millipore, 0.22 μm). Ethical approval was obtained from the University of Auckland Human Participants Ethics Committee (025496) for use of blank human plasma for the method development, which was supplied by New Zealand Blood Services (Auckland, New Zealand).

### 2.2 Chromatographic conditions

An Agilent 1200 HPLC machine (Agilent Technologies, Germany) equipped with photo diode array detector was employed for method development and quantitative analysis. The samples were analysed on Cosmosil 5 C18 column (250 × 4.6 mm, 5 μm) from Phenomenex, New Zealand. The mobile phase consisted of 10 mM potassium phosphate buffer, pH 7.4 and acetonitrile in a ratio of 75:25 v/v with a flow rate of 1 mL/min. The injection volume was 20 μL and detection was carried out at 270 nm.

### 2.3 Protocol for extraction of diazoxide from plasma samples

For extraction of diazoxide, 50 μL plasma was vortex mixed with 650 μL acetonitrile at 2000 rotations per minute (RPM) for 5 min before being centrifuged at 6708×*g*, and 650 μL supernatant was collected in a new Eppendorf tube. Another 300 μL of acetonitrile was added to the residue and the above process was repeated. Subsequently, 300 μL of supernatant was combined with the supernatant collected in the first step. The supernatant was evaporated to dryness with a gentle nitrogen stream at 40 °C and the residue was reconstituted with 50 μL of 0.05 M potassium hydroxide (KOH) solution. The reconstituted samples were centrifuged at 12045×*g* and 20 μL of supernatant was injected for HPLC evaluation. The extraction recovery was determined using Eq. (1).

(1) $$\text{Recovery}\;\left(\%\right)=\frac{\text{Peak}\;\text{area}\;\text{of}\;\text{analyte}\;\text{extracted}\;\text{in}\;\text{plasma}}{\text{Peak}\;\text{area}\;\text{of}\;\text{analyte}\;\text{in}\;\text{solution}}\times 100$$

### 2.4 Method validation

The extraction protocol was validated using the developed stability-indicating HPLC method according to bioanalytical method validation guidelines [14]. Forced degradation studies were conducted to determine whether the method was stability indicating (see Supplementary Material).

#### 2.4.1 Preparation of calibration standards

Ten-fold concentrated solutions of diazoxide dissolved in 0.05 M KOH were used to spike the blank human plasma to generate triplicates of calibration standards at 0.2, 0.5, 1, 2, 5, 10, 20 and 40 μg/mL. Similarly, another set of triplicate spiked plasma samples were prepared at concentrations of 2.5 15 and 30 μg/mL and were used as the quality controls (QC). The plasma calibration standards and QC samples were freshly prepared on each day of validation.

#### 2.4.2 Sensitivity

The sensitivity of the method was determined from the signal-to-noise ratio of the lowest non-zero calibrator. The lower limit of quantitation was defined as a signal-to noise ratio >10 [14].

#### 2.4.3 Linearity

Freshly prepared plasma samples ranging from 0.2 to 40 μg/mL were used to generate the calibration standards for three consecutive days. Peak area values of the prepared standard calibrators were plotted versus the nominal concentration and the co-efficient of regression was determined to establish linearity of the method.

#### 2.4.4 Accuracy and precision

The intra- and inter-run (three consecutive days) accuracy of the assay was assessed via the plasma QC samples in triplicate. Accuracy was considered acceptable if the mean QC concentration was within 15% of the nominal concentration.

Precision is closeness of the repeated measurement at same concentration. It was determined from the relative standard deviation (RSD) of triplicate plasma QC samples in a single run and over three consecutive daily runs. Precision was considered acceptable if the RSD was <15%.

#### 2.4.5 Specificity

Chromatograms of blank plasma, spiked plasma and standard diazoxide solution were compared to identify any interference due to the biological sample matrix. The developed method should be specific to determine the analyte of interest in the presence of other cross-reacting molecules.

#### 2.4.6 Freeze-thaw, autosampler and long-term storage stability

A separate set of triplicate spiked ‘stability’ plasma samples at 0.5, 2.5 and 15 μg/mL were used to determine pre- and post-extraction stability of diazoxide. To determine pre-extraction freeze-thaw stability, the spiked plasma samples were thawed from −20 °C to ambient temperature every 24 h. Test samples were then withdrawn, extracted, and analysed for stability. Long-term storage stability of plasma samples was determined to mimic the duration between sample collection and analysis during the clinical study. For this, spiked plasma samples were stored at −20 °C and a sample was withdrawn and analysed every month for up to three months. Post-extraction stability of diazoxide was determined following storage of the extracted plasma samples in an autosampler without temperature control for up to 48 h.

### 2.5 Stability protocol of diazoxide suspensions

**Fig. 1:**
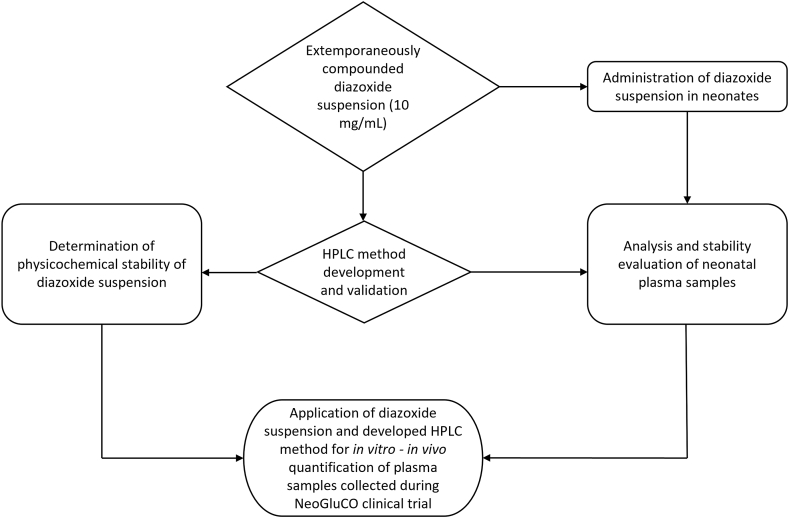
Overview of study methodology, outlining the process of developing and validating the HPLC method to quantify diazoxide in both the extemporaneously compounded suspension and to determine diazoxide in neonatal plasma samples.

For the stability evaluation, twelve 10 mg/mL diazoxide suspensions (50 mL) were extemporaneously prepared using Ora Blend® SF as the vehicle. The relationship between the developed HPLC method and the compounded diazoxide suspensions are outlined in Fig. 1. The suspensions were prepared at the Middlemore Hospital Pharmacy (Counties Manukau Health, Auckland, New Zealand). The contents of 5 × 100 mg capsules were emptied into the mortar and triturated using a small amount of the vehicle to form a smooth paste. The mixture was then transferred into a graduated cylinder and appropriate dilution was prepared using the Ora Blend® SF. The prepared suspensions were then transferred into an amber coloured container and stored protected from light at 2–8 °C or 25 °C, 60% relative humidity (RH) (n = 3 for each condition). A separate set of suspensions were stored in 50 mL stoppered volumetric cylinders at each storage condition to determine sedimentation volume ratio (SVR). Samples were withdrawn on days 0, 7, 14, 21, 28 and 35 to evaluate the physical and chemical stability of the suspensions as below.

#### 2.5.1 Physical stability

A representative sample of 3 mL was withdrawn at each time point and the suspension was analysed for physical stability by organoleptic evaluation and SVR.

##### 2.5.1.1 Organoleptic evaluation

Suspensions were evaluated for colour and consistency on each day of analysis by visual observation against a dark background. Odour was also evaluated at each time point.

##### 2.5.1.2 Sedimentation volume ratio

SVR was used to quantify the degree of flocculation of the suspension. On each day of analysis, all suspensions in the volumetric cylinders were redispersed uniformly by manually inverting the cylinder at 180° four times and the volume of suspension (Vo) was determined. The suspensions were left undisturbed for 24 h and then the volume of sediment (Vu) was measured. SVR was calculated using Eq. (2).

(2) $$SVR=Volume\;of\;sediment\;\left(V_{u}\right)/Volume\;of\;suspension\;\left(V_{0}\right)$$

##### 2.5.1.3 Ease of redispersion

On each day of analysis, the volumetric cylinders in both storage conditions were inverted and the number of 180° inversions needed to achieve a uniform suspension were recorded.

#### 2.5.2 Chemical stability

##### 2.5.2.1 pH

The pH of each suspension was measured using a micro pH electrode (Seven Easy, Mettler, Toledo). The pH meter was calibrated prior to use on each day of analysis with standard buffer solutions at pH 4, 7 and 10.

##### 2.5.2.2 Quantification of diazoxide

At each time point, a 100 μL sample was withdrawn from the suspension and diluted with 900 μL of 0.05 M KOH solution in water: methanol (50:50). The sample was then vortex mixed at 2000 RPM for 1 min and centrifuged at 10621×*g* for 10 min. Following that, 20 μL of supernatant was collected in another Eppendorf tube and was diluted with 980 μL of 0.05 M KOH solution in water: methanol (50:50). From the resulting solution, 10 μL was injected in the HPLC system for quantitative analysis of diazoxide.

### 2.6 Osmolality of diazoxide suspensions

The osmolality of diazoxide suspensions, prepared as described above, was measured using the vapor pressure method (Vapro osmometer 5600, EliTech Group, New Zealand). Samples were first diluted ten times using MilliQ water to ensure measurement within the calibration range of the instrument and tested in triplicate. For comparison, placebo samples were tested in the same way. These comprised Ora Blend® SF with a small amount of corn starch added to maintain blinding ahead of the clinical trial.

### 2.7 Application of method to clinical samples

The validated HPLC method was applied to clinical and placebo samples (six each) collected in the NeoGluCO Study. Neonates received a loading dose of 0.5 ml/kg of the diazoxide suspension (5 mg/kg), followed by a 12-hourly maintenance dose of 0.15 ml/kg (1.5 mg/kg), or equal volume of placebo. Heparinised blood was collected before the third or fourth study maintenance dose and centrifuged at 2000×*g* and 4 °C for 10 min. Plasma was separated and stored in 150 μL aliquots at −80 °C. Samples were thawed for at least 30 min prior to analysis and diazoxide was quantified using the plasma extraction and HPLC methods described in Section 2.3 above. The NeoGluCO Study, which is ongoing, received ethical approval from the New Zealand Health and Disability Ethics Committee (19CEN189). Blinding of investigators and participants to trial intervention allocation was maintained.

## 3 Results and discussion

### 3.1 Extraction recovery

The two-step extraction protocol resulted in almost complete extraction of diazoxide from the plasma QC samples, with a recovery of more than 99% (Table 1).

**Table 1:** Accuracy, precision and extraction recovery of the plasma diazoxide quality control (QC) samples at concentrations of 2.5, 15 and 30 μg/mL. RSD, relative standard deviation. Data are mean (SD).

Plasma QC diazoxide concentration (μg/mL)	Intra-day (n = 3)		Inter-day (n = 9)		Extraction recovery (%) (n = 9)
	Accuracy (%)	RSD (%)	Accuracy (%)	RSD (%)	
2.5	101.3 (0.5)	0.5	99.3 (2.4)	2.5	101.2 (2.1)
15	103.6 (5.1)	4.9	101.2 (5.2)	5.1	99.6 (2.6)
30	97.4 (0.5)	0.5	95.6 (3.6)	3.7	98.4 (4.2)

### 3.2 Method validation

A rapid, sensitive, and reproducible extraction protocol was validated using the HPLC method, which eluted diazoxide at a mean (SD) of 8.9 (0.3) min, as described below.

#### 3.2.1 Sensitivity

The developed method was sensitive to detect diazoxide in plasma samples at the concentration level of 0.2 μg/mL with a signal-to-noise ratio of 12:1. The limit of detection was determined to be 0.02 μg/mL.

#### 3.2.2 Linearity

The developed method was linear within the range of 0.2–40 μg/mL on three consecutive days. The mean (SD) regression coefficients were y = 50.350 (3.188) x – 8.469 (4.736), with the R^2^ value > 0.9994.

#### 3.2.3 Accuracy and precision

The developed method was accurate at all three plasma QC concentrations, with triplicate mean concentrations within 5% of the nominal concentration, both for a single run and over three consecutive daily runs (Table 1). The method was also found to be precise with RSD ≤6% over three consecutive daily runs.

#### 3.2.4 Specificity

**Fig. 2:**
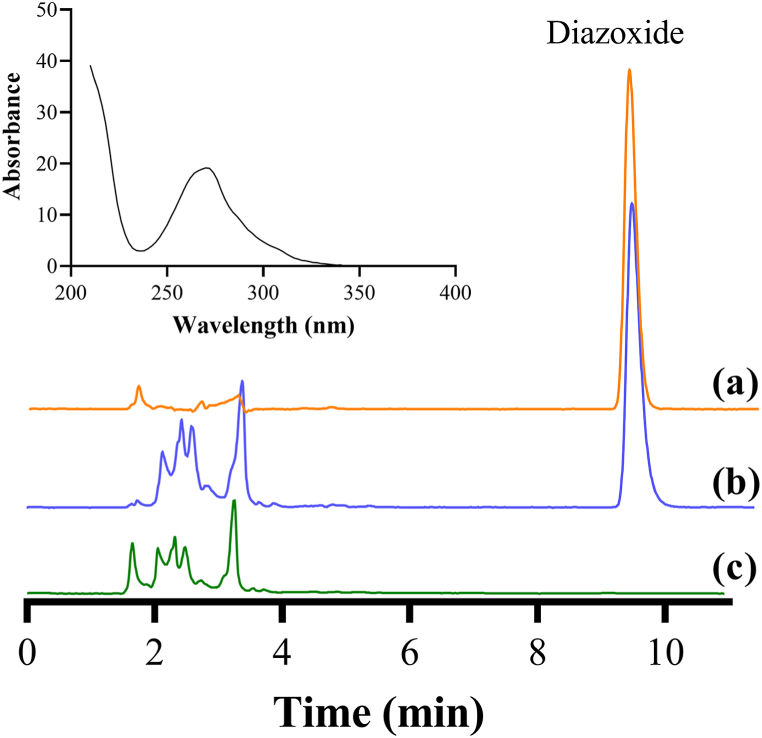
Overlaid chromatograms of (a) 10 μg/mL standard diazoxide solution, (b) plasma spiked with diazoxide at 10 μg/mL and (c) blank plasma, showing the specificity of the developed method at 270 nm. This wavelength was selected following a spectra scan using a photo diode array detector (inset).

The method was specific to detect diazoxide in human plasma, as demonstrated by the overlaid chromatograms of diazoxide sample solutions at 10 μg/mL, spiked plasma and blank plasma (Fig. 2 a - c).

#### 3.2.5 Freeze-thaw, auto sampler and storage stability

The plasma samples spiked with diazoxide were found to remain stable for two freeze-thaw cycles, with more than 98% diazoxide remaining at all sample concentrations (0.5, 2.5, 15 μg/mL). Following a third freeze-thaw cycle, drug concentration in the 2.5 and 15 μg/mL samples dropped to 2.2 and 13.9 μg/mL, representing 88% and 94% of the spiked amount, respectively. Upon long term storage at −20 °C, samples were stable for three months. After the diazoxide had been extracted from spiked plasma samples (post-extraction), it was stable for 48 h. This indicated stability for the duration of analysis. The stability of diazoxide samples are summarised in Table 2.

**Table 2:** Freeze-thaw, autosampler and long-term storage stability of plasma diazoxide samples at 0.5, 2.5 and 15 μg/mL. RSD, relative standard deviation. Data are mean ± RSD of n = 3 samples. Pre-extraction means plasma spiked with diazoxide, post-extraction indicates diazoxide has been extracted from the spiked plasma samples.

Concentration (μg/mL)	Freeze thaw, two cycles (pre-extraction)		Auto sampler stability, 48 h (post-extraction)		Long term storage at −20 °C, three months (pre-extraction)	
	Drug remaining (%)	RSD (%)	Drug remaining (%)	RSD (%)	Drug remaining (%)	RSD (%)
0.5	98.9	2.4	106.3	10.9	94.2	1.2
2.5	105.7	4.1	111.8	6.0	101.1	3.7
15	100.7	3.0	103.7	4.6	97.5	1.3

Together, these results indicate that storage conditions of −20 °C for up to three months is appropriate for clinical samples, and that clinical samples can withstand two freeze-thaw cycles and be maintained in the non-controlled environment of the auto-sampler for 48 h.

### 3.3 Stability evaluation of diazoxide suspensions

#### 3.3.1 Physical stability

Extemporaneously prepared diazoxide suspensions were physically stable when stored at 2–8 °C and 25 °C, 60% RH for up to 35 days, as outlined below. Stability in both conditions is important as medications may be variable stored at room temperature or refrigerated in the neonatal nursery environment.

##### 3.3.1.1 Organoleptic evaluation

**Fig. 3:**
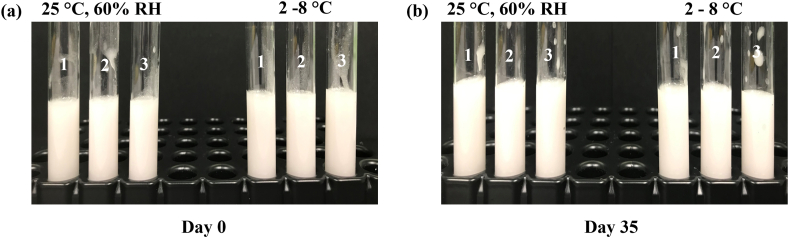
Images showing absence of colour change in extemporaneously prepared diazoxide suspensions (10 mg/mL) when kept at 2–8 °C and 25 °C, 60% RH at (a) day 0, and (b) day 35.

No organoleptic changes were observed up to 35 days at either storage condition, with all samples maintaining their initial opaque light pink colour and berry/citrus scent (Fig. 3). All the suspensions maintained uniform consistency over the study period and no phase separation was identified. This differed to findings observed in a previous study that investigated diazoxide suspensions compounded with Oral Mix as the vehicle, where sedimentation was observed after 7 days [10]. According to Stokes’ law, sedimentation velocity is inversely proportional to the viscosity of the liquid and indeed Oral Mix is reported to have a lower viscosity than the Ora Blend used in this study [15]. The physical stability of the intervention in a randomised clinical trial is important as it contributes to effective blinding of allocation. The consistent organoleptic properties of the diazoxide suspensions over time is a key reason why the active intervention and placebo used in the NeoGluCO Study demonstrated very high sensory equivalence when tetrad testing was used amongst neonatal staff [9].

##### 3.3.1.2 Sedimentation volume ratio (SVR)

The SVR was >0.99 for all the suspensions at all time points, which is close to the ideal flocculation value for suspensions of 1.0, indicating good deflocculation. Furthermore, the suspensions at both storage conditions were easy to redisperse, requiring only 3–4 inversions indicating ease and uniformity of dose administration.

#### 3.3.2 Chemical stability

Extemporaneously prepared diazoxide suspensions were chemically stable when stored at 2–8 °C and 25 °C, 60% RH for up to 35 days, as outlined below.

##### 3.3.2.1 pH

The pH of the diazoxide suspensions was stable over the 35-day period. At the end of study, mean (SD) pH of all the suspensions was 4.24 (0.01) and 4.24 (0.03) at 2–8 °C and 25 °C, 60% RH, respectively. No significant change in the suspension pH was observed compared with the same at day 0.

##### 3.3.2.2 Quantification of diazoxide using HPLC

**Fig. 4:**
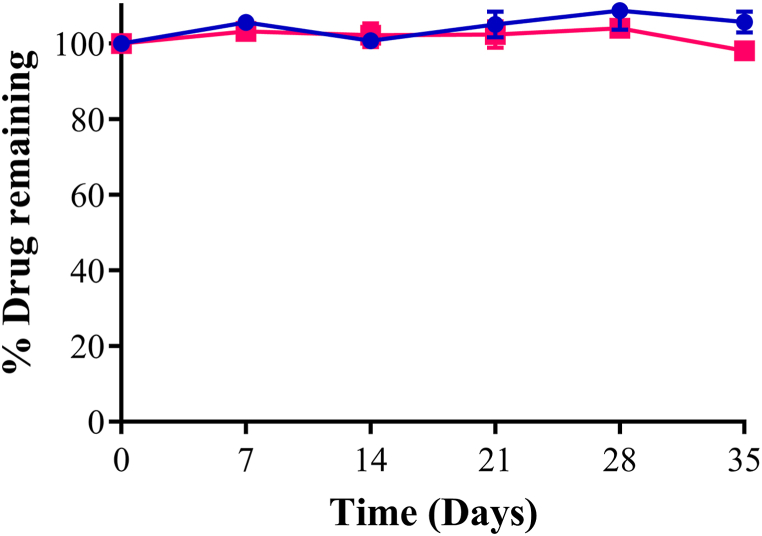
Chemical stability of extemporaneously prepared diazoxide suspensions (10 mg/mL) at two storage conditions; (●) 2–8 °C and (□) 25 °C, 60% relative humidity.

Over the 35-day period, >98% diazoxide remained in the suspensions for both storage conditions (Fig. 4). The initial (Day 0) mean (SD) concentration of the suspensions to be stored at 2–8 °C and 25 °C, 60% RH were 9.46 (0.29) mg/mL and 9.99 (0.13) mg/mL. At the end of stability evaluation, mean (SD) diazoxide remaining was 105.7 (2.8)% and 98 (0.4)% at 2–8 °C and 25 °C, 60% RH, respectively. According to the United States Pharmacopoeia, the presence of >90% of the drug following storage indicates formulation stability [16]. The findings of this study were similar to that reported by Friciu et al. [10], who reported stability of diazoxide in Oral Mix products for 90 days. These results indicate that storage at room temperature is appropriate and allow the conservative assignment of a 30-day expiry, which aligns with other extemporaneously compounded oral liquid preparations in New Zealand. This will enable batch preparation of diazoxide suspension and reduce potential treatment delays due to compounding time.

### 3.4 Osmolality of diazoxide suspensions

The mean (SD) osmolality of the suspensions were 1308 (80.1) mOsm/kg and 1042 (42.3) mOsm/kg for diazoxide and placebo, respectively. These values are higher than that recommended by the American Academy of Pediatrics [17], but lower than that reported for many other medicines commonly used in neonates [[18], [19], [20]].

### 3.5 Application of method to clinical samples

**Fig. 5:**
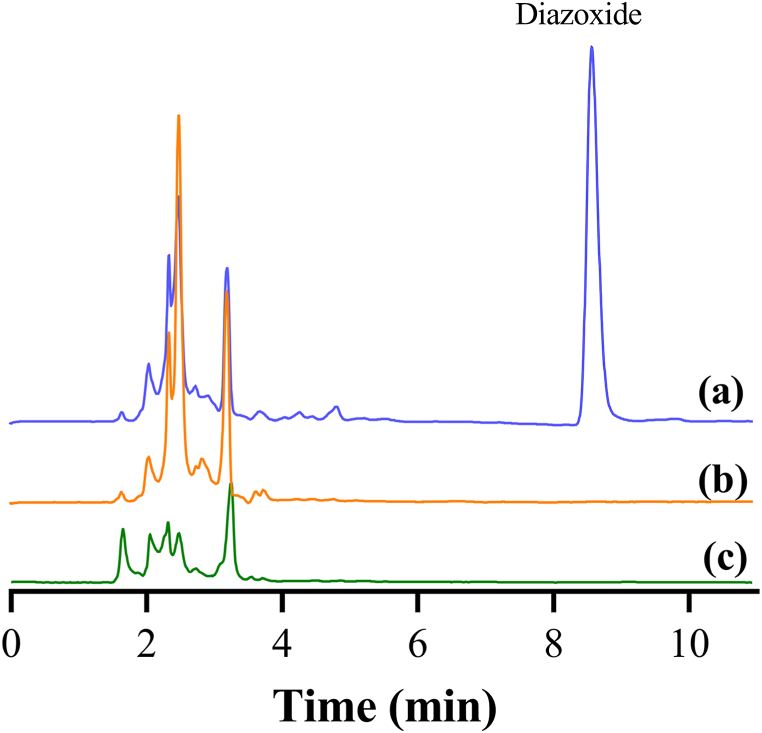
Representative chromatograms showing efficiency of the applied method to differentiate between clinical plasma samples from neonates allocated to (a) diazoxide or (b) placebo group in the NeoGluCO Study, compared with (c) blank plasma sample.

The characteristics of neonates in whom clinical samples were obtained are summarised in Table 3. The mean (SD) concentration of diazoxide in the clinical samples (n = 6) was 23.5 (2.8) μg/mL, which is the mid-point of the calibration curve, and this demonstrates that the assay is appropriate for quantifying diazoxide in clinical samples. Moreover, no diazoxide peak was detected in the samples from the neonates treated with placebo (n = 6), confirming that no contamination occurred during the compounding process (Fig. 5 a - c). With an accurate and precise diazoxide assay available, further research can now be undertaken to characterise the pharmacokinetic profile of diazoxide in preterm and term neonates in the early newborn period and to relate this to pharmacodynamic response. Our clinical sample included mostly well early-term infants who commenced treatment on the first day after birth. More information is needed about dosing in preterm infants, those with renal impairment, and when treatment is continued beyond the first week after birth, when both renal and hepatic clearance may change.

**Table 3:** Characteristics of neonates receiving diazoxide (n = 6) from whom clinical samples were obtained.

Case #	1	2	3	4	5	6
Gestation length (completed week)	38	36	37	36	40	37
Birthweight category	SGA	LGA	SGA	AGA	LGA	LGA
Age diazoxide commenced (hours)	17	10	31	18	22	8
Sex	Male	Male	Male	Female	Male	Male
Plasma creatinine concentration prior to commencing diazoxide (μmol/L)	75	91	79	126	55	–
Number of maintenances doses after loading and before sampling	3	2	3	2	2	3
Plasma diazoxide concentration (μg/mL)	25.6	22.3	27.7	19.7	23.6	22.2

SGA, small for gestational age; LGA, large for gestational age; AGA, appropriate for gestational age.

## 4 Conclusion

A rapid, reproducible and sensitive extraction protocol was developed and validated for accurate and precise quantification of diazoxide using a stability indicating HPLC method. The developed method was linear over the range of 0.2–40 μg/mL with R^2^ > 0.999 and extraction recovery >94% of all the plasma samples. Extemporaneously compounded diazoxide suspensions were physically and chemically stable for up to 35 days, both at 2–8 °C and 25 °C, 60% RH, indicating that a 30-day expiry at room temperature is appropriate. The developed extraction protocol was successfully applied to determine the diazoxide concentration in neonatal plasma samples collected during the NeoGluCO Study, and has future potential for determining the pharmacokinetic profile of diazoxide for the treatment of transitional neonatal hypoglycaemia in neonates.

## Author contribution statement

Trusha J Purohit: Don Laing: Performed the experiments; Analysed and interpreted the data; Wrote the paper.

Christopher JD McKinlay: Sara Hanning: Conceived and designed the experiments; Analysed and interpreted the data; Contributed reagents, materials, analysis tools or data; Wrote the paper.

Jane M Alsweiler: Conceived and designed the experiments; Contributed reagents, materials, analysis tools or data; Wrote the paper.

## Data availability statement

Data will be made available on request.

## Funding

This work is supported by the 10.13039/501100001537University of Auckland (Early Career Research Award, CJDM) and 10.13039/501100001505Health Research Council of New Zealand (20/651).

## Ethics statement

This study received ethical approval from the New Zealand Health and Disability Ethics Committee (19CEN189).

## Declaration of competing interest

The authors declare that they have no known competing financial interests or personal relationships that could have appeared to influence the work reported in this paper.
