# Supplementary material for: Development and clinical application of a stability-indicating chromatography technique for the quantification of diazoxide
Source: Heliyon. 2023 Sep 13;9(9):e20101. doi: 10.1016/j.heliyon.2023.e20101 (PMC10559840; doi:10.1016/j.heliyon.2023.e20101)
Supplement: Multimedia component 1 [file mmc1.docx]

Development and clinical application of a stability-indicating chromatography technique for the quantification of diazoxide: Supplementary data

This supplementary information outlines the method validation for the quantification of extemporaneously compounded diazoxide suspension, including forced degradation of diazoxide to assess the stability-indicating nature of the assay. For method validation of the quantification of diazoxide following plasma extraction, please refer to the main manuscript.

# Material and methods

## Materials

Please refer to the materials section of the manuscript.

## Method validation for *in vitro* quantitation of diazoxide

The method was validated according to ICH guidelines for analytical method validation[1]

### Preparation of calibration standards and quality control (QC) samples

A standard stock solution of 1 mg/mL diazoxide was prepared using 0.05 M KOH solution: methanol (50:50 v/v). Seven calibration standards were prepared in triplicate within the range of 0.2 – 50 µg/mL from the 1 mg/mL stock solution. Three QC samples were prepared from a completely different standard solution at concentrations of 0.4, 10 and 30 µg/mL.

### Linearity

The linearity was determined based on the calibration curve generated by plotting peak area versus concentration values.

### Sensitivity

The sensitivity of the method was determined by multiple injections of serially diluted diazoxide solution and evaluation of the signal to noise ratio. The limit of detection and quantitation of the developed method was determined based on signal to noise ratio values above 3.3 and 10, respectively.

### Accuracy and precision

Accuracy of the method indicates closeness of the determined concentrations to the true values of the concentrations. This was analysed by multiple injections of QC (n = 9) samples.

Precision is a measure of degree of agreement between the measurements upon multiple sample injections at different time and days. This was determined by inter-day and intra-day evaluation of the QC samples (n = 9) that were injected over three consecutive days.

### Specificity

The specificity of the method was determined by injecting different concentrations of diazoxide extracted from an extemporaneously made suspension and were compared with the standard diazoxide solutions made at the same concentration level.

### Forced degradation analysis

To determine the stability indicating nature of the HPLC assay, diazoxide was subjected to different hydrolysis (pH 2 or 10), photolytic and oxidative stress conditions to generate the degradation products [2-4]. Diazoxide solutions were prepared at 200 µg/mL concentration using 0.1 mM hydrochloric acid (HCl), 0.1 mM sodium hydroxide (NaOH) water and 0.3% hydrogen peroxide (H_2_O_2_). Duplicates of the acidic, basic and water samples were kept at 60 °C, while the other set of duplicates in water were exposed to UV light ranging from 320-400 nm at 25 °C. The oxidative stress degradation was carried out at 25°C protected from light. To determine the specificity of the method, samples were withdrawn at predetermined time intervals, appropriately diluted and subjected to HPLC analysis to evaluate the ability of the method to specifically resolve diazoxide from its degradation products. The degradation was carried out for up to seven days or until 10% degradation of diazoxide was observed, whichever was earlier.

## Method validation

### Linearity


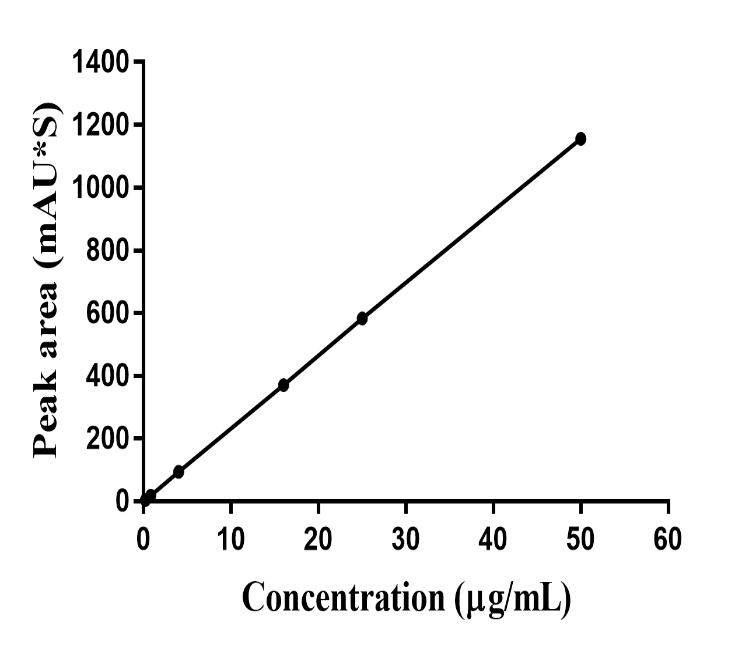
The HPLC method was found linear within the range of 0.2 – 50 µg/mL with the R^2^ value > 0.999 (y = 23.125x + 0.8976) (Figure 1).

**Figure 1** Calibration curve within the concentration range of 0.2 - 50 µg/mL (n= 3). Data represented as mean ± SD.

### Sensitivity

The method was found sensitive and the limit of quantification and limit of detection were found to be 0.2µg/mL and 0.014 µg/mL, respectively.

### Accuracy and precision

The developed method was found to be accurate and precise. The accuracy of the method was determined at three concentrations of 0.4, 10, 30 µg/mL. The accuracy of the method was found within 93-100 % and the relative standard deviation (RSD) values were found below 4 for all three concentrations. The results of accuracy and precision have been summarised in Table 2.

**Table 1** Accuracy and precision of diazoxide compounded suspensions at a concentration level of 0.4, 10, 30 µg/mL. RSD, relative standard deviation. Data are presented as mean (SD)

| **Theoretical concentration (µg/ml)** | **Intra-day (n=3)** | | | **Inter-day (n=9)** | | |
| --- | --- | --- | --- | --- | --- | --- |
|  | **Experimental concentration (µg/ml)** | **RSD**  **(%)** | **Accuracy**  **(%)** | **Experimental concentration (µg/ml)** | **RSD**  **(%)** | **Accuracy**  **(%)** |
| 0.4 | 0.37 (0.01) | 2.21 | 92.3 (2.3) | 0.37 (0.00) | 1.14 | 92.9 (1.1) |
| 10 | 9.85 (0.09) | 0.95 | 98.5 (0.9) | 9.92 (0.07) | 0.72 | 99.2 (0.7) |
| 30 | 29.74 (0.06) | 0.19 | 99.1 (0.2) | 30.22 (0.46) | 1.51 | 100.7 (1.5) |

### Specificity

The method was found to be specific and was able to determine a diazoxide peak from the suspension matrix. The chromatograms of diazoxide standard solution and diazoxide suspension have been compared in Figure 2.


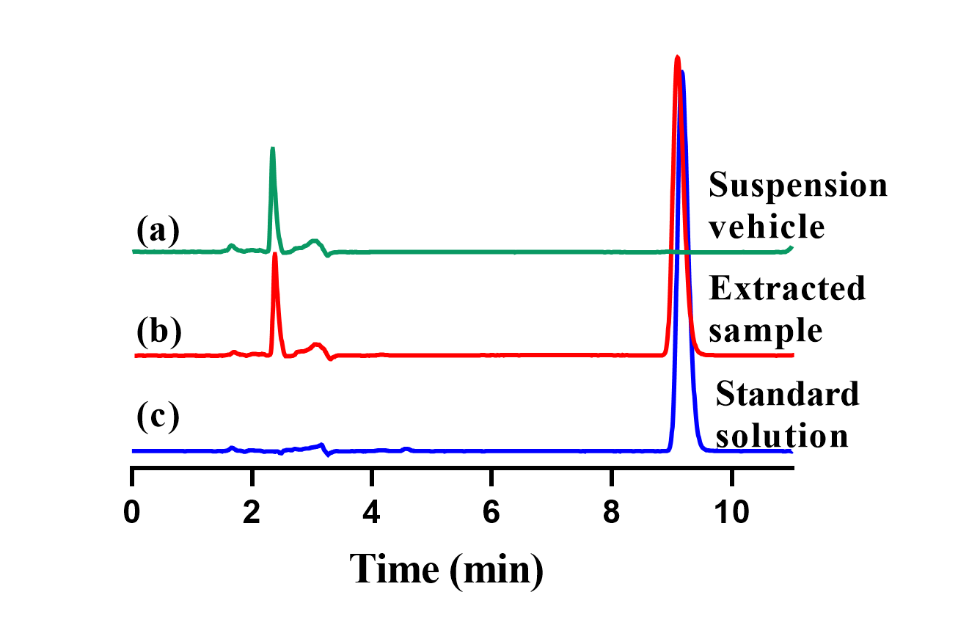


Figure 2 Overlaid chromatograms of (a) blank suspension vehicle (OB SF), (b) 20 µg/mL extracted diazoxide samples from suspension, (c) standard diazoxide solution at 25 µg/mL, showing the specificity of the developed method at 270 nm.

### Forced degradation of diazoxide

Forcedd degradation studies confirmed the stability indicating nature of the developed method. The developed method was found specific enough to satisfactorily resolve the degradation products from diazoxide (Figure 3). Although small variation in retention time (within ± 0.3 min) was observed, there were no interfering degradation product peaks that could affect analysis.


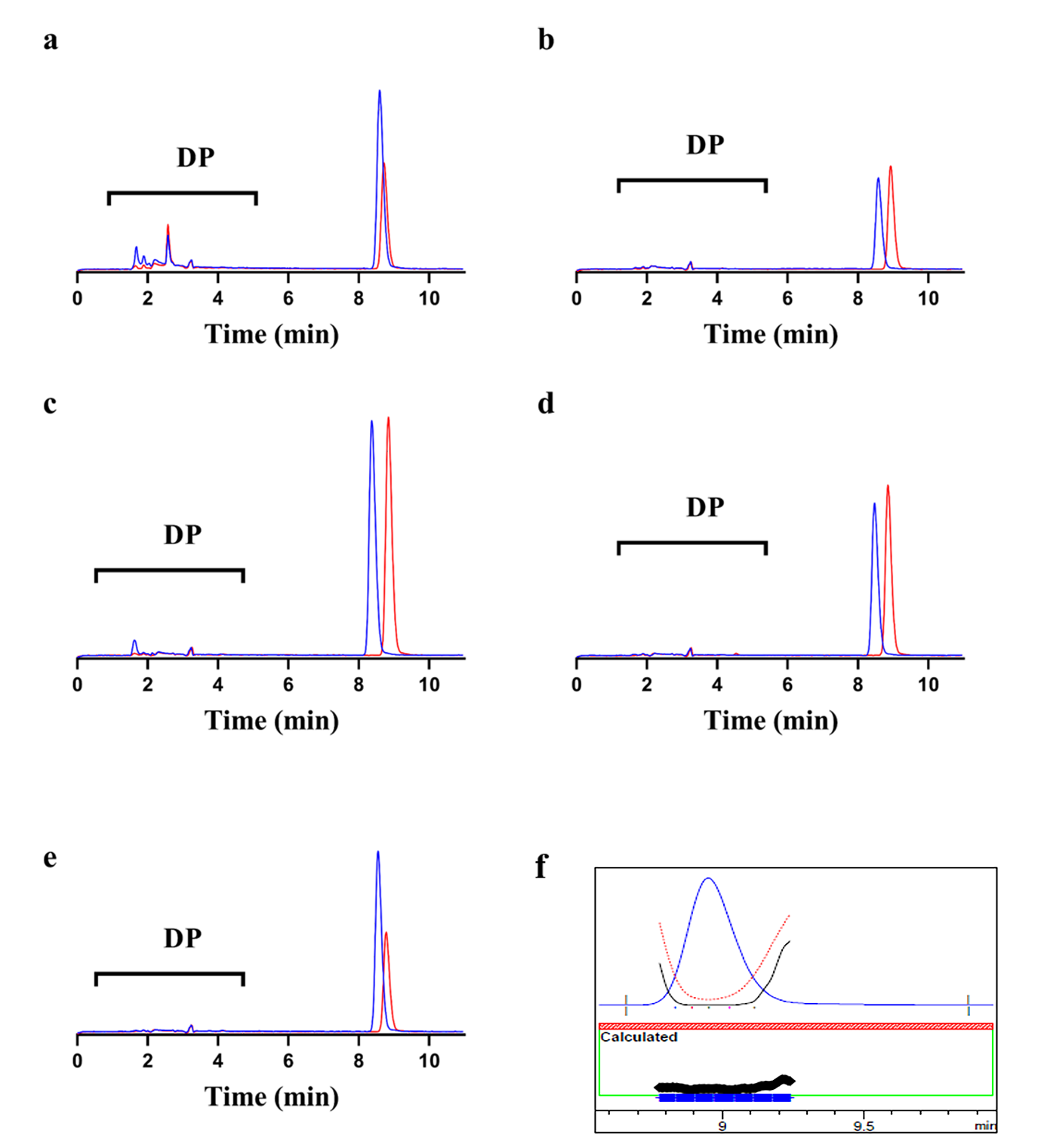


**Figure 3** Chromatograms representative of the specificity of the method to determine diazoxide from its degradation products generated under stress conditions: (a) 0.% H_2_O_2_, (b) 0.1 M HCl, (b) 0.1 M NaOH, (c) UV and (d) control. (f) represents peak purity of the diazoxide peak in the chromatograms. Concentration of diazoxide at t = 0 (blue line) and t = day 4 (red line). DP – degradation products.

**References**

[1] ICH topic Q2 (R1) Validation of analytical procedures: Text and methodology: European Medicines Agency; 1995 [cited 2017 22 September]. Available from: <https://www.ema.europa.eu/en/documents/scientific-guideline/ich-q-2-r1-validation-analytical-procedures-text-methodology-step-5_en.pdf>

[2] Ngwa G. Forced degradation as an integral part of HPLC stability-indicating method development. Drug Deliv Technol. 2010;10(5):56-9.

[3] Shah BP, Jain S, Prajapati KK, Mansuri NY. Stability indicating HPLC method development: A Review. Int J Pharm Sci Res. 2012;3(9):2978.

[4] Blessy M, Patel RD, Prajapati PN, Agrawal YK. Development of forced degradation and stability indicating studies of drugs-A review. J Pharm Anal. 2014;4(3):159-65.
